# The neurofucntional abnormalities of temporal gyrus underly impaired sensory attenuation in schizophrenia during action-outcome contingent paradigm

**DOI:** 10.1038/s41598-025-07950-2

**Published:** 2025-07-20

**Authors:** Weihao Huang, Jing Shi, Yunhui Ma, Yucong Zhang, Yongqian Wang, Xuteng Wang, Yaling Wen, Shaokun Zhao, Shuping Tan, Zhiren Wang

**Affiliations:** Beijing Huilongguan Hospital, Peking University Huilongguan Clinical Medical School, Beijing, 100096 China

**Keywords:** Schizophrenia, Sensory attenuation, Temporal gyrus, Task-based functional magnetic resonance imaging, Action-outcome contingent paradigm, Schizophrenia, Cortex, Sensory processing

## Abstract

Previous research suggests that individuals with schizophrenia may exhibit impairments in sensory attenuation. This neurocognitive process is defined as reduced neural responses in sensory cortices to self-generated actions compared with externally triggered sensory inputs. However, the specific neuroimaging association between sensory attenuation deficits and schizophrenia has not been fully established. To elucidate the neuroimaging signatures of these impairments, the present study employed a modified action-outcome contingent paradigm combined with a task-based functional magnetic resonance imaging in 20 individuals with schizophrenia and 21 matched healthy controls. Participants completed voluntary (active) and experimenter-administered (passive) button-press tasks respectively, both eliciting auditory feedback, to isolate the neural correlates of sensory attenuation. Neuroimaging analysis revealed characteristic abnormalities in neural activity within the left middle temporal gyrus/superior temporal gyrus of patients. First, compared to the healthy controls, absence of activation attenuation (passive-active) was observed in these regions under active conditions. This phenomenon suggests a specific impairment in the ability to discriminate self-generated stimuli. Second, when identical auditory stimuli were passively received, significantly lower baseline activation levels within these regions were found in patients than in healthy controls. This task-specific characteristic highlights impaired detection and evaluation of environmentally driven stimuli in patients.

## Introduction

The nosological entity of “schizophrenia” was originally introduced by Eugen Bleuler, who observed that individuals with this disorder often experience “the most diverse alterations of the self”, including a fragmentation of self-awareness and a loss of the ability to perceive or guide one’s own actions and thoughts ^1^. To better understand the potential disruptions of self-awareness in schizophrenia, relevant research ^2^ has identified two aberrant mechanisms underlying self-disorders in schizophrenia:

1. *Hyperreflexivity* A pathologically heightened self-monitoring state, wherein intrinsic self-features are overly concerned and misattributed to the external environment. In schizophrenia, auditory hallucinations are thought to stem from such heightened awareness. Some researchers hypothesize that individuals with schizophrenia exhibit excessive attention to internal experiences, such as inner speech, and erroneously ascribe its origin to external agents ^3^.
2. *Diminished self-affection* A weakening of the subjective sense of selfhood. The term “self-affection” is not related to a specific sensory modality, but refers to a fundamental awareness of being the agent of one’s own actions and existence. In schizophrenia, patients may perceive their spontaneous thoughts, actions, or perceptions as being controlled by external forces. This inability to recognize the self as the causal agent is a prototypical manifestation of diminished self-affection ^4^ and clinically manifested as “passivity experiences” ^5^.

Notably, whether hyper-attribution of self-features (hyperreflexivity) or hypo-recognition of self-agency (diminished self-affection), convergent evidence from multimodal neuroimaging, such as event-related potential (ERP) ^6^, diffusion tensor imaging (DTI) ^7^, functional magnetic resonance imaging (fMRI) ^8^ and ocular motor paradigms ^9^, suggests that these various physiological changes in schizophrenia involve impaired sensory attenuation mechanisms responsible for distinguishing self-generated versus external sensory inputs.

Within the central nervous system, sensory attenuation serves as a critical mechanism for distinguishing between exafferent sensory inputs originating from environmental interactions and reafferent inputs generated by self-initiated actions ^10^. As elucidated by Pickering and Garrod et al. in their model of target-directed hand movement ^11^, this discriminative process involves three sequential stages:

1. *Motor command execution* Motor commands are issued to initiate an action and a corresponding actual sensory feedback (E).
2. *Predictive outcome generation* Concurrently, an efference copy of the motor command generates a predicted sensory outcome (P), enabling the agent to anticipate the sensory consequences of the action ^12^.
3. *Comparison and attribution* The comparison between P and E determines perceptual attribution:

Case 1 (Prediction Error Scenario, P ≠ E): Discrepancy between predicted outcome and actual feedback triggers an error signal via sensory neuron activation, prompting the individual to highlight the mismatch and make corrective adjustments in subsequent movements. For instance, if the predicted hand position deviates rightward from the actual trajectory, this prediction error will be encoded to induce proportional rightward compensation in subsequent movement iterations ^13^.

Case 2 (Prediction Matching Scenario, P = E): Congruence between predicted outcome and actual feedback induces sensory attenuation, a neurocognitive phenomenon characterized by diminished attention to self-generated sensations ^14^. This process allows the individual to infer that the sensory experience resulted from their own intentional action, thereby reinforcing a sense of agency (SoA) and volitional control over the experience ^15^.

However, previous studies have consistently revealed that, compared to healthy populations, individuals with schizophrenia exhibit marked impairments in attributing SoA to self-generated sensory experiences ^16^. Additionally, this deficit is primarily attributable to the diminished subjective experience of sensory attenuation—that is, the failure to attenuate the perceived intensity of sensations resulting from self-generated actions. Electrophysiological research has also provided converging evidence for this impairment across standardized paradigms. For instance, in classical auditory event-related paradigms, self-initiated auditory stimuli in healthy controls typically elicit a reliable suppression of the N1 component, whereas individuals with schizophrenia display aberrantly reduced N1 suppression in response to the same stimuli ^17^.

Crucially, this defective sensory attenuation is believed to underlie the mechanism of impaired discrimination between “self” and “non-self” in schizophrenia ^18^. Furthermore, this deficit can severely impair patients’ understanding of their own actions (e.g., passivity experiences), perception of internal states (e.g., auditory hallucinations), and interaction with the external environment (e.g., negative symptoms). However, the underlying neural mechanisms of these perceptual impairments remain incompletely understood, posing significant challenges for clinical intervention.

Consequently, a growing body of neuroimaging research has aimed to delineate the neuroanatomical substrates associated with sensory attenuation. For instance, studies in healthy populations suggest that this function primarily involves two complementary neural systems^19^: (1) a feedforward network centered on the cerebellum, responsible for mediating real-time comparisons between predicted outcome and actual feedback; (2) a feedback network encompassing the superior temporal gyrus (STG), middle temporal gyrus (MTG), and insula, involved in detecting exogenous stimulus features to provide real-time feedback reference for the predictive outcomes generated by the feedforward network.

However, multimodal neuroimaging studies have systematically verified that individuals with schizophrenia show dysfunctional functional connectivity (FC) patterns at multiple nodes within these two core systems:

1. Within the feedforward network, reduced FC strength between the cerebellum and thalamus was found in patients by resting-state fMRI ^20^. Structural integrity deficits in white matter tracts connecting the cortex, cerebellum, and thalamus were also confirmed by DTI ^21,22^. Notably, the cortico-cerebello-thalamo-cortical circuit is considered central to sensory attenuation mechanisms ^23,24^: Multimodal input signals from sensory cortices are received by the cerebellum in this circuit. These signals are then filtered and relayed by the thalamus. Finally, error-correction signals are fed back to the primary sensory cortex to optimize behavioral output.
2. Within the feedback network, decreased connectivity between the right anterior insula and the right STG was observed in patients using resting-state fMRI ^25^. This phenomenon may be linked to the misattribution of auditory hallucination. Additionally, reduced information flow between the insula and MTG was further correlated with worsening cognitive symptoms, as shown by whole-brain magnetoencephalography studies ^26^.

Although an increasing number of studies have implicated sensory attenuation deficits in schizophrenia stemming from feedforward-feedback network dyscoordination, the exact neuroimaging signatures remain elusive. To address this gap, the present study employed a modified action-outcome contingent paradigm in conjunction with task-based fMRI. Participants were instructed to perform voluntary (active) and experimenter-administered (passive) button-presses respectively, with auditory feedback provided in both conditions. By contrasting neural responses across active and passive conditions, this design allows for the precise discrimination of self-initiated versus externally induced sensory inputs, effectively isolating the neural correlates of sensory attenuation while controlling for confounding action-related variables.

Taken together, we hypothesized that, compared to healthy controls, individuals with schizophrenia would exhibit the following features: (1) defective activation attenuation in key brain regions associated with sensory attenuation (e.g., cerebellum, thalamus, STG, MTG, and insula) during self-generated actions; (2) disrupted FC patterns between these regions; (3) significant associations between the degree of sensory attenuation deficits and the severity of clinical symptoms. These hypotheses aim to unravel the neurofunctional basis of impaired sensory attenuation in schizophrenia and establish neurofunctional correlates of its clinical manifestations.

## Methods

### Participants

Participants were recruited between August 2023 and the time of statistical analysis through offline advertisements at Beijing Huilongguan Hospital. A total of 22 individuals with schizophrenia and 22 healthy controls matched for gender, age, and educational level were initially enrolled. Due to excessive head motion (translation > 3 mm or rotation > 3°), imaging data from 2 patients and 1 control were excluded. Ultimately, data from 20 patients and 21 healthy controls were included in the final analysis.

The diagnosis of schizophrenia was confirmed by two experienced licensed psychiatrists based on the criteria of the Diagnostic and Statistical Manual of Mental Disorders, Fifth Edition (DSM-5). Patients met the diagnostic criteria for schizophrenia and did not meet criteria for any other major psychiatric disorders, including neurodevelopmental disorders, other psychotic disorders, bipolar and related disorders, depressive disorders, trauma- and stressor-related disorders, substance-related and addictive disorders, neurocognitive disorders.

Healthy controls and their first-degree relatives were assessed to ensure no current or past diagnosis of any major psychiatric disorder according to DSM-5 criteria, including neurodevelopmental disorders, schizophrenia spectrum and other psychotic disorders, bipolar and related disorders, depressive disorders, trauma- and stressor-related disorders, substance-related and addictive disorders, neurocognitive disorders,

All participants met the following inclusion criteria: (1) right-handedness confirmed by the Edinburgh handedness inventory; (2) aged 18–55 years without gender restriction; (3) at least junior high school education with normal intelligence and ability to comprehend and complete study-related assessments and procedures; (4) absence of severe or unstable physical illness, particularly seizure-related conditions, such as epilepsy or other disorders that may induce seizures (e.g., neurological diseases or severe head trauma); (5) no contraindications for MRI scanning, including electronic/metal implants (e.g., pacemakers, neurostimulators, implanted pumps, cochlear implants, vascular clips, metallic prostheses, or permanent eyeliner) and no history of claustrophobia; (6) absence of comorbid known or suspected borderline or antisocial personality disorders, or any other psychiatric conditions of sufficient severity to interfere with study participation..

This study was approved by the Ethics Committee of Beijing Huilongguan Hospital. All participants and their legal guardians were fully informed of the purpose and procedures of the study and voluntarily provided written informed consent. To minimize the risk of coercion, informed consent was obtained by research staff rather than psychiatric clinicians involved in patients’ treating. All study procedures have been performed in accordance with the Declaration of Helsinki.

### Action-outcome contingent paradigm

The effect of sensory attenuation is frequently assessed using ERP techniques combined with the action-outcome contingent paradigm. Typically, this paradigm consists of three core experimental conditions to systematically examine the interaction between action execution and sensory feedback ^27^:

1. *Action-outcome condition* Participants are instructed to voluntarily perform a repetitive action, such as pressing a button, tapping a key, or moving a finger. Each action elicits a perceptible sensory consequence, such as auditory feedback.
2. *Stimulus-only condition* Participants remain still and passively receive a series of stimuli without executing movements. The effect of sensory attenuation is generally defined as the differences of ERPs between action-outcome and stimulus-only conditions ^28^.
3. *Action-only condition* A supplementary control condition employed in prior ERP studies, where participants voluntarily repeat actions as in the action-outcome condition but receive no contingent sensory outcomes. The ERPs recorded in this condition are subtracted from those in the action-outcome condition to isolate action-contingent sensory modulation ^28^.

Although the action-outcome contingent paradigm holds significant value in sensory attenuation research, the application of this classical design requires consideration of its methodological limitations. Traditionally, two key design aspects require refinement:

First, significant cognitive load imbalance exists between experimental conditions. Specifically, both the action-outcome and action-only conditions require participants to actively perform designated actions. In contrast, the stimulus-only condition involves purely passive stimulus reception. This asymmetry of task may lead to differences in attentional resource between conditions. Consequently, systematic bias may be introduced into the comparison of neural activity across conditions. Therefore, recent studies have improved condition comparability. Cognitive operational complexity has been balanced across conditions, and attention-monitoring tasks (e.g., oddball detection task) have been added ^29,30^.

Second, the paradigm’s homogeneity assumption regarding the effects of action lacks sufficient validation. Its core presupposition is that the motor execution process is neurophysiologically equivalent in the action-outcome and action-only conditions. However, supporting evidence remains insufficient. Direct validation of motor cortex activity consistency between these conditions using electrophysiological techniques is lacking. Furthermore, empirical data comparing neuroimaging signatures of sensory attenuation-related brain regions using fMRI are also scarce. This gap between theoretical presupposition and empirical foundation may compromise the validity of causal inferences regarding the neurofunctional characteristics of sensory attenuation ^28^.

**Fig. 1:**
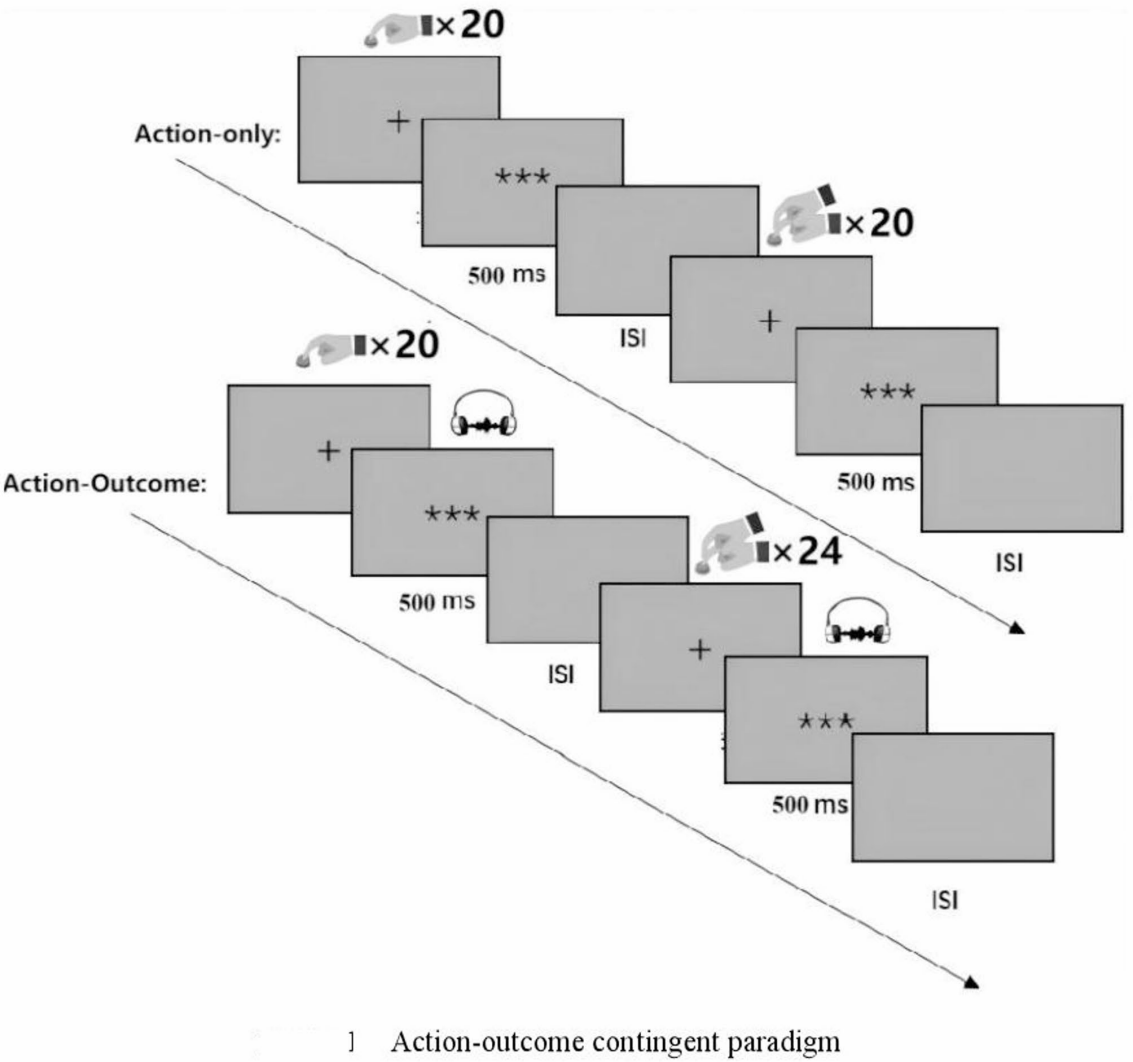
Action-outcome contingent paradigm.

To address these limitations, the present study implemented a modified action-outcome contingent paradigm wherein the traditional stimulus-only condition was replaced with a passive button-press condition (Fig. 1). In this condition, participants were instructed to remain still and keep their right index finger on the button while the experimenter manually pressed participants’ right index finger, thus maintaining biomechanical equivalence while eliminating voluntary action components. This methodological adaptation optimizes control over confounding action-related variables between active and passive conditions.

Consequently, the formal experimental protocol comprised two counterbalanced conditions:

1. *Active condition* Participants were instructed to press a button using their right index finger at a self-chosen moment (typically within 4 s) following the appearance of a fixation cross (“ + ”). This temporal constraint was designed to elicit a well-prepared, self-initiated action rather than a direct response to the fixation cue.
2. *Passive condition* Participants were required to keep their finger stationary. The researcher then manually pressed the participant’s right index finger to complete the key press within the same time window (within 4 s after fixation onset). Autonomous motor intention was thereby eliminated, effectively distinguishing biological effects between intentional modulation and mechanical movement.

Critically, a staged experimental design was employed to precisely dissociate neural responses to spontaneous versus externally triggered sensory inputs. This design facilitated the investigation of sensory attenuation while controlling for confounding motor variables:

The first stage specifically examined neurofunctional characteristics under the action-only condition to clarify confounding effects of motor variables. Experimental validity was ensured through the following core designs:

1. Two movement modes were established, participants either actively pressed a button with their right index finger or received passive finger movements administered by an experimenter (20 trials each). Each trial lasted 6 s, followed by a 4-s rest period, totaling 6 min and 40 s. Additionally, the order of active and passive conditions was randomized across participants to balance the effects of sequence.
2. The auditory feedback channel was completely blocked throughout the action-only stage. Moreover, this stage was consistently implemented before the action-outcome stage to eliminate the potential influence of anticipatory outcome effects on neural activity in the action-only condition. This was crucial because, in the action-outcome condition, button-presses (both active and passive) were associated with sensory consequences.

The subsequent stage specifically examined neurofunctional characteristics under the action-outcome condition. This examination aimed to contrast neural processing differences between self-initiated and passively triggered stimuli. Therefore, a standardized feedback mechanism was established to enhance result comparability. Specifically, a precisely time-locked system was implemented for both conditions: Following button-press triggering, an acoustic stimulus (0.5 kHz pure tone or 1 kHz white noise) was presented within a ≤ 1 ms time window and persisted for 500 ms. Furthermore, all acoustic stimuli were delivered via a customized MRI-compatible audio system.

Consistent with the action-only condition, an active–passive dual-condition paradigm was also constructed for the action-outcome condition to enhance research validity. Specifically, the experiment comprised 44 trials (20 active + 24 passive). A between-subject randomization strategy was adopted for order balancing. Consequently, systematic interference from the sequence of active/passive conditions on research was prevented. Moreover, the settings of temporal parameter were aligned with the action-only condition. Each trial lasted 6 s, followed by a 4-s inter-trial rest period. Total experimental duration was controlled at 7 min and 20 s. Thus, by unifying temporal parameters and dual-condition paradigm of movement modes, interference from extraneous variables was effectively controlled. Differences in experimental effects could therefore be attributed to the core characteristics of the action execution mode.

To address the imbalance of attentional load in the passive condition, participants were informed beforehand to perform an oddball detection task (500 ms, 1 kHz noise) during the passive phase. Operationally, four oddball stimuli (1 kHz white noise, 500 ms) were randomly triggered by four button-presses, and participants were required to press a button using their left index finger upon detecting the oddball stimuli. Attention levels were thereby effectively maintained. It should be noted that all trials containing oddball stimuli were excluded from subsequent data analysis.

### Acquisition and preprocessing of fMRI data

All task-based fMRI data were acquired at Beijing Huilongguan Hospital Neuroimaging Center using a 3.0 T Siemens Prisma scanner with a 12-channel head coil. Whole-brain coverage was achieved via gradient-echo planar imaging (EPI) sequence with 32 axial slices (5 mm thickness) acquired in ascending interleaved acquisition (foot-to-head, slice-skipping). Imaging parameters included: voxel size = 3.8 × 3.8 × 5 mm^3^, field of view = 240 mm, repetition time = 2000 ms, echo time = 30 ms, flip angle = 70°. Each participant completed 84 trials yielding 420 whole-brain EPI images across a 14-min scanning session.

To facilitate co-registration of functional and structural images, high-resolution T1-weighted structural images were obtained using a magnetization-prepared rapid gradient-echo sequence with the same scanner and identical scanner configuration: voxel size = 1 × 1 × 1 mm^3^, field of view = 256 mm, repetition time = 2000 ms, echo time = 2.28 ms, flip angle = 9°.

All fMRI data were preprocessed using RESTplus v1.30_20240508 (http://www.restfmri.net/forum/RESTplus), a task-based fMRI batch preprocessing toolkit built on SPM12 (https://www.fil.ion.ucl.ac.uk/spm/software/spm12) and operated via MATLAB R2022b (https://www.mathworks.cn/products/matlab.html). The “Pipeline” module of RESTplus was used for batch preprocessing and the following preprocessing steps were applied:

1. *Realignment* EPI images were realigned to the median image of each time series to correct for slice-timing and head motion.
2. *Coregistration* Each participant’s T1-weighted structural images were coregistered to their corresponding mean EPI images.
3. *Segmentation* Gray matter, white matter, and cerebrospinal fluid were segmented using the New Segment toolbox in SPM12.
4. *Normalization* Segmented structural images were normalized to the Montreal Neurological Institute (MNI) standard space and the transformation was applied to the EPI images (resampled at 3 × 3 × 3 mm^3^ resolution). Systematic visual inspection was performed across all participants to verify registration accuracy and exclude spatial artifacts after normalization.
5. *Smooth* A 6 mm full-width half-maximum Gaussian kernel was applied to spatially smooth the normalized EPI images.

### Statistical analysis

First, preprocessed EPI images were subjected to first-level analysis for each participant. Each experimental condition was modeled as an independent regressor, and six head motion parameters were included as nuisance covariates. All regressors were convolved with the canonical hemodynamic response function.

Second, a 2 (group: patients vs. controls) × 2 (condition: active vs. passive) mixed-design ANOVA was conducted to estimate main effects of group, main effects of condition, and group × condition interaction effects. For brain regions showing significant interaction effects, post-hoc analysis were performed to determine activation differences between groups and conditions. The clusters with significant interactions were then defined as seed regions (6-mm radius spheres) for psychophysiological interaction (PPI) analysis, which aimed to explore condition-dependent (active − passive) FC differences. Independent samples *t*-tests were used to assess between-group differences in FC. Statistical significance for blood oxygen level dependent signal time courses was assessed at cluster-level threshold of family-wise error (FWE) correction, *P* < 0.05.

Finally, the interaction-significant clusters were subsequently defined as regions of interest (ROIs, 6-mm radius spheres) to extract the activation difference between conditions (passive − active) for each schizophrenia patient. These values were correlated with clinical variables including illness duration, chlorpromazine equivalents dose, positive and negative syndrome scale (PANSS) scores (total score, and positive/negative/general psychopathology scores) using SPSS 22.0 (https://www.ibm.com/spss).

For each result, the peak voxel coordinates (*x*, *y*, *z* in MNI space), cluster size (number of voxels), the multiple comparison correction method, and statistical values (*t* or *F* values) were reported. All imaging results were visualized using MRIcroGL (https://www.nitrc.org/projects/mricrogl/).

## Results

### Demographic characteristics and clinical profiles

There were no significant differences between the schizophrenia group and the healthy control group in demographic variables, including sex, age, and years of education. The schizophrenia group had an average illness duration of 6. 64 years (SD = 3. 91). All patients were receiving antipsychotic treatment at the time of the study, and all medications were atypical antipsychotics, including: amisulpride (n = 3), aripiprazole (n = 6), blonanserin (n = 1), clozapine (n = 5), lurasidone (n = 3), olanzapine (n = 7), paliperidone (n = 6), perospirone (n = 1), and risperidone (n = 8). The daily dosage of antipsychotics was converted into chlorpromazine equivalents using published conversion tables ^31^, with a mean dose of 378.89 mg/day (SD = 215.78 mg/day) (Table 1).

**Table 1:** Demographic characteristics and clinical profiles.

Characteristic	Patients Mean (SD)	Controls Mean (SD)
*Demographic characteristics*		
N, male: female	12:10	12:10
Age, years	23.72 (3.91)	22. 58 (3.04)
Years of education	14.44 (2.01)	13. 89 (2.05)
*Clinical profiles*		
Duration of illness, years	6.64 (3.91)	——
Chlorpromazine equivalents, mg/d	378.89 (215.78)	——
PANSS		
Total score	88.00 (4.77)	——
Positive scale	24.39 (1.58)	——
Negative scale	23.83 (3.91)	——
General psychopathology scale	39.78 (2.92)	——

Symptom severity and classification in patients was assessed using the PANSS. The mean total PANSS score was 88.00 (SD = 4.77). The mean scores for positive scale, negative scale, and general psychopathology scale were 24.39 (SD = 1.58), 23.83 (SD = 3.91), and 39.78 (SD = 2.92) respectively (Table 1).

### Task-related brain activation differences under the action-only condition between groups

ANOVA revealed no significant main effects of condition or group × condition interactions, but a significant main effect of group was observed.

The main effect of group showed significant activation differences between groups in the following brain regions: nucleus of the thalamus, fusiform gyrus, inferior occipital gyrus, inferior frontal gyrus, insula, central operculum, STG, putamen, precentral gyrus, cingulate cortex, postcentral gyrus, angular gyrus and transverse temporal gyrus (Table 2).

**Table 2:** 2 × 2 mixed-design ANOVA data for the action-only condition.

Group	Region	Cluster (voxels)	Peak voxel coordinate				*P*_FWE_ (cluster-level)
			x	y	z	F	
Main effect of group							
Cluster 1	Fusiform_L	266	− 27	− 66	− 9	173.47	< 0.001
	Thal_VPL_L	——	− 21	− 21	9	51.76	——
Cluster 2	Fusiform_R	262	27	− 66	− 3	113.62	< 0.001
	Occipital_Inf_R	——	36	− 63	− 9	48	——
Cluster 3	Insula_L	74	− 33	21	6	82.95	0. 001
	Frontal_Inf_Orb_2_L	——	− 39	30	− 3	23.53	——
Cluster 4	Thal_VPL_R	36	21	− 24	6	60.4	0. 03
	Thal_PuM_R	——	9	− 27	0	56.24	——
Cluster 5	Putamen_L	261	− 27	− 18	9	55.43	< 0. 001
	Temporal_Sup_L	——	− 54	− 15	0	37.33	——
	Rolandic_Oper_L	——	− 36	− 24	18	35.97	——
Cluster 6	Precentral_L	82	− 42	0	33	47.84	0. 001
	Frontal_Inf_Oper_L	——	− 45	15	18	21.55	——
Cluster 7	Cingulate_Mid_L	172	0	− 30	48	41.04	< 0. 001
Cluster 8	Parietal_Inf_L	70	− 30	− 42	48	36.35	0. 002
	Postcentral_L	——	− 33	− 36	39	31.95	——
Cluster 9	Temporal_Sup_R	157	54	− 27	9	31.95	< 0. 001
	Heschl_R	——	45	− 24	12	28.61	——
	Insula_R	——	36	− 30	18	27.8	——

### Task-related brain activation differences under the action-outcome condition between groups

ANOVA revealed a significant group × condition interaction identified in the left MTG (*F* = 24.13) and left STG (*F* = 36.35; cluster-level *P*_FWE_ < 0.001) (Table 3). These two regions were subsequently defined as ROIs (6-mm radius spheres centered at MNI coordinates: left MTG [− 60, − 21, − 6] and left STG [− 54, − 9, − 6]) for post-hoc analysis.

**Fig. 2:**
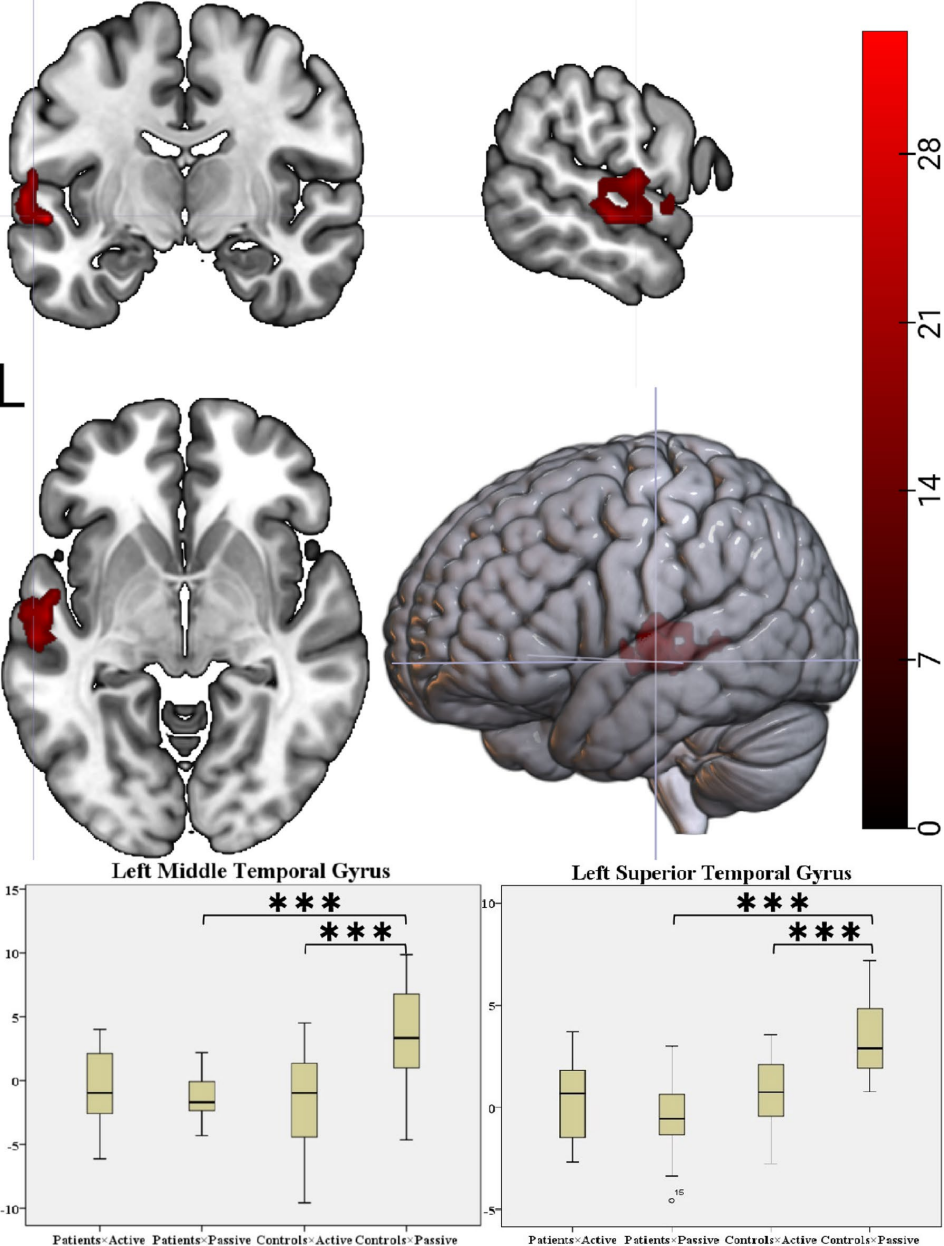
Task-related brain activation differences under the action-outcome condition between groups. ANOVA revealed a significant group × condition interaction identified in the left middle temporal gyrus and left superior temporal gyrus. Post-hoc analysis revealed that controls demonstrated hypoactivation in the left middle temporal gyrus and left superior temporal gyrus while comparing active to passive conditions—consistent with intact sensory attenuation mechanisms. However, patients exhibited a dual pathology in these regions: (1) absence of attenuation during active actions, and (2) hypoactivation during passive conditions relative to controls.

1. *Within-group contrasts* Controls exhibited significant hypoactivation in the left MTG (*t* =  − 6.12, *P* < 0.001) and left STG (*t* =  − 7.00, *P* < 0.001) while comparing active conditions to passive conditions. However, no significant activation differences between conditions were found in patients for either region (Fig. 2) (Table 4).
2. *Between-group contrasts* During passive conditions, patients demonstrated significant hypoactivation relative to controls in the left MTG (*t* =  − 4.52, *P* < 0.001) and left STG (*t* =  − 6.53, *P* < 0.001). No significant between-group differences were observed in either region during active conditions (Fig. 2) (Table 4).

**Table 3:** 2 × 2 mixed-design ANOVA data for the action-outcome condition.

Group	Region	Cluster (voxels)	Peak voxel coordinate				*P*_FWE_ (cluster-level)
			x	y	z	F	
Interaction							
Cluster 1	Temporal_Sup_L	117	− 54	− 9	− 6	36.35	< 0.001
	Temporal_Mid_L	——	− 60	− 21	− 6	24.13	——
Main Effect of Condition							
Cluster 1	Cerebellum_6_L	2810	− 12	− 69	− 12	72.28	< 0.001
	Fusiform_L	——	− 24	− 72	− 15	70.58	——
	Calcarine_L	——	− 12	− 69	12	49.11	——
Cluster 2	Temporal_Mid_R	79	57	− 33	− 6	48.89	0.002
Cluster 3	Postcentral_R	85	36	− 21	45	38.6	0.001
	Precentral_R	——	48	− 12	39	20.63	——
Cluster 4	Cerebellum_6_R	38	33	− 42	− 24	36.19	0.04
	Fusiform_R	——	42	− 39	− 15	19.45	——
Cluster 5	Temporal_Mid_L	50	− 51	− 45	12	30.34	0.016
	SupraMarginal_L	——	− 45	− 48	27	20.86	——
Cluster 6	Temporal_Sup_R	45	36	− 30	12	30.05	0.023
	SupraMarginal_R	——	45	− 36	21	21.15	——
Cluster 7	Postcentral_L	100	− 48	− 15	36	25.67	0.001
	Rolandic_Oper_L	——	− 57	0	12	24.25	——
	Precentral_L	——	− 45	− 6	36	21.44	——
*Main effect of group*							
Cluster 1	Rolandic_Oper_L	5117	− 36	− 33	15	315.85	< 0.001
Cluster 2	ACC_sup_L	203	− 3	21	27	58.99	< 0.001
	Cingulate_Mid_R	——	6	21	33	33.36	——
	Frontal_Sup_2_L	——	− 12	18	42	31.77	——
Cluster 3	Caudate_R	64	18	− 12	21	55.96	0.006
Cluster 4	Insula_R	125	36	27	6	50.4	< 0.001
Cluster 5	Precuneus_L	478	− 3	− 36	57	37.73	< 0.001
	Cingulate_Mid_L	——	− 6	− 9	39	37.58	——

**Table 4:** Post-hoc analysis for the action-outcome condition.

Factor	Comparison	Mean Deviation (SD)	t	*P*
Temporal_Sup_L				
Controls	Active − Passive	− 2.84 (1.77)	− 7.00	< 0.001***
Patients	Active − Passive	0.82 (1.82)	1.91	0.073
Passive	Patients − Controls	− 3.87 (0.59)	− 6.53	< 0.001***
Active	Patients − Controls	− 0.21 (0.65)	− 0.32	0. 751
Temporal_Mid_L				
Controls	Active − Passive	− 5.01 (3.57)	− 6.12	< 0.001***
Patients	Active − Passive	0.70 (3.31)	0.90	0.381
Passive	Patients − Controls	− 4.61 (1.02)	− 4.52	< 0.001***
Active	Patients − Controls	1.10 (1.13)	0.97	0.339

#### Task-related functional connectivity differences in key brain regions between groups

PPI analysis was performed using the left MTG (MNI: −60, −21, −6) and left STG (MNI: −54, −9, −6) as seed regions (6-mm radius spheres) to examine condition-specific (active–passive) FC differences. Independent samples *t*-tests were conducted to assess group differences:

Compared to controls, patients showed reduced task-related FC between the left MTG and the right caudate nucleus (cluster size: 20 voxels; MNI: 15, −6, 18) across conditions (*t* = 4.54, *P* < 0.001 uncorrected).

#### Correlations between task-related activation differences in key brain regions and clinical variables in patients

Task-related activation differences (passive–active) in the left MTG (MNI: −60, −21, −6) and left STG (MNI: −54, −9, −6) were extracted from each subject in patients based on the defined ROIs (6-mm radius spheres centered at MNI coordinates), and Pearson correlation analysis was performed:

1. No significant correlations were found between activation differences in either region and illness duration or chlorpromazine equivalent dosage.
2. Activation differences in the left MTG were negatively correlated with negative scale scores (*r* =  − 0.55, *P* = 0.017 uncorrected). No significant correlations were observed with PANSS total scores, positive scale scores, or general psychopathology scale scores. For the left STG, activation differences exhibited no significant correlations with PANSS total scores and any subscale (Table 5).

**Table 5:** Correlation analysis for the action-outcome condition and PANSS.

PANSS	r	P	*P*_FWE_
Temporal_Sup_L			
Duration of illness	− 0.19	0.452	1
Chlorpromazine equivalents	0.42	0.085	1
Total score	0.01	0.981	1
Positive scale	0.19	0.447	1
Negative scale	0.03	0.906	1
General psychopathology scale	− 0.13	0.597	1
Temporal_Mid_L			
Duration of illness	− 0.15	0.556	1
Chlorpromazine equivalents	− 0.07	0.778	1
Total score	− 0.07	0.790	1
Positive scale	0.37	0.127	1
Negative scale	− 0.55	0.017*	0.204
General psychopathology scale	0.43	0.075	0.9

It should be noted that after correcting for multiple comparisons (Bonferroni correction), the relationship between the left MTG and PANSS negative symptom scale was no longer statistically significant. Consequently, the stability of this association could not be confirmed and further correlation analysis of the PANSS negative symptom subscales was not conducted in this study.

## Discussion

This study utilized an action-outcome contingent paradigm integrated with task-based fMRI to systematically elucidate the neurofunctional characteristics of sensory attenuation deficits in schizophrenia. The key findings revealed that controls demonstrated hypoactivation in the left MTG/STG while comparing active to passive conditions—consistent with intact sensory attenuation mechanisms. However, patients failed to exhibit a comparable pattern in the left MTG/STG, suggesting reduced modulation rather than a reversed response. Furthermore, in passive conditions, patients showed significant hypoactivation in these regions relative to controls, suggesting compromised processing of detecting exogenous stimulus features.

Previous studies on the neural mechanisms underlying SoA abnormalities in schizophrenia present a multidimensional theoretical framework ^32^. Specifically, some evidence supports enhanced self-attribution ^33,34^ and intentional binding effects ^35^ in schizophrenia. Conversely, other studies have also identified significantly weakened neural markers related to SoA in patients ^36,37^. Therefore, the mechanisms underlying aberrant SoA experiences in schizophrenia are complex. These experiences cannot be attributed solely to a tendency towards either excessive or diminished self-attribution. However, this theoretical controversy may stem from an imbalance between the feedforward prediction generation and sensory feedback integration ^38,39^.

Therefore, to address the above theoretical divergence, a pattern of neurofunctional abnormality was revealed in this study using a hierarchical analysis strategy:

1. During the action-only condition phase, the main effect and group × condition interaction were analyzed. No statistically significant difference in regional brain activation was detected between active and passive key-press conditions. This key finding provided a critical premise for the subsequent analysis of the action-outcome condition. Specifically, interference from motor execution itself on neural signals was effectively excluded.
2. An interaction model (group × condition) was applied to analyze the action-outcome condition: first, no significant between-group differences emerged in neural activation during active conditions; second, compared to controls, patients exhibited significant hypoactivation in the left STG/MTG during passive conditions; third, and most critically, in patients, this hypoactivation abolished the activation difference pattern across task conditions, a neuroimaging signature that was conspicuously observed in controls.

Notably, the MTG region identified in this study (MNI coordinates: −60, −21, −6) shows high spatial overlap with key target regions in previous SoA research ^40,41^. For example, recent neuroimaging studies suggest that the MTG may be involved in constructing the dynamic neural representation of SoA. This involvement is achieved by monitoring temporal discrepancies between predictive signals and actual sensory input ^42^. Collectively, this evidence supports the clinical finding of this study: MTG dysfunction may represent a core mechanism underlying SoA abnormalities in schizophrenia.

Critically, the experimental design of this study was explicitly guided by the sensory attenuation theoretical framework. Specifically, during the passive key-press condition, auditory stimuli were triggered by an external program. This condition strictly adheres to the SoA paradigm definition for non-self-generated stimuli. Consequently, when stimuli were self-initiated by active actions, the characteristic attenuation of activation (passive-active) observed within the left MTG of control subjects can be considered a neuroimaging marker. This marker reflects the successful suppression of sensory feedback by feedforward signaling—consistent with sensory attenuation theory for normal SoA neural mechanisms. However, a significantly abnormal pattern was observed within this region of patients: The typical activation attenuation phenomenon was absent. This suggests a specific impairment in the ability to discriminate self-generated stimuli ^43^.

These findings provide new insights for reconciling existing theoretical paradoxes. Specifically, functional imbalance in the MTG may simultaneously affect two key components of the sensory attenuation mechanism—feedforward prediction generation and sensory feedback integration: Impaired feedforward prediction generation generation prevents patients from suppressing the perceptual consequences of their own actions. Meanwhile, abnormal sensory feedback integration affects the precise parsing of perceptual information from environmental input. This dual mechanism defect may reconcile seemingly contradictory phenomena in previous studies: Behavioral manifestations of enhanced self-attribution are observed, while objective evidence of weakened SoA neuroimaging markers is also present.

Furthermore, in controls, the left STG also demonstrated inter-condition attenuation while comparing active conditions to passive conditions, consistent with previous task-based fMRI findings ^44^—reduced activation in the STG during self-initiated speech compared to passive listening conditions. This finding was extended by the present study using a modified action-outcome contingent paradigm: STG involvement in distinguishing active/passive non-verbal auditory stimuli was confirmed and this cross-modal functional consistency suggests that the STG may serve as a key node within the sensory attenuation neural circuit.

Therefore, the pattern of neurofunctional abnormality revealed in this study provides neuroimaging evidence for the sensory attenuation deficits in schizophrenia: the simultaneous absence of activation attenuation within the left MTG/STG of patients suggests that neurofunctional abnormalities in these regions may represent a core neuroimaging marker for sensory attenuation deficits and disrupted functional integration within the STG-MTG-insula feedback network, a tripartite system responsible for detecting exogenous stimulus features ^19^.

Correspondingly, significant hypoactivation within these regions was indeed observed in patients when identical stimuli were passively received. However, no significant between-group differences in activation under active conditions was detected between the patients and the healthy controls. This “active preservation-passive suppression” dissociation pattern carries greater pathological significance. It is suggested that sensory attenuation deficits in patients may not originate from dysfunction in motor execution itself. Instead, impaired detection and evaluation of environmentally driven stimuli are highlighted.

Finally, exploratory analysis suggested a potential negative correlation between the magnitude of activation attenuation in the left MTG and the severity of negative symptoms. This finding suggests that sensory attenuation, or functional abnormalities in the MTG, may be linked to negative symptoms. Additionally, reduced FC was observed between the left MTG and the right caudate nucleus in patients. Therefore, abnormal FC between the left MTG and the right caudate nucleus may play a role in the pathological mechanisms of schizophrenia or in neural circuits related to sensory attenuation deficits.

Despite these significant findings, the study has several limitations:

1. Although medication dosages were standardized to chlorpromazine equivalents, all patients were on antipsychotic medication, and the potential pharmacological effects may interfere with the interpretation of neural activation.
2. The cross-sectional experimental design limits our ability to draw causal conclusions about the relationship between sensory attenuation deficits and clinical symptoms. Future studies should incorporate longitudinal research or intervention experiments to further validate these findings, with an emphasis on subgroup analysis based on symptom dimensions.
3. The PPI and correlation analysis results were not corrected for multiple comparisons, and further studies with larger sample sizes are needed to verify the reproducibility of these findings.

In conclusion, this study systematically revealed the neurofunctional characteristics underlying sensory attenuation deficits in schizophrenia: First, the functional properties of the left MTG/STG as a key brain region for sensory attenuation were identified. Reduced effects of activation attenuation (passive-active) were observed within this region of patients. This finding suggests a specific impairment in the discrimination of self-generated stimuli in patients. Furthermore, a dissociated pattern characterized by “active preservation-passive suppression” was revealed in patients. Such characteristics highlight impaired detection and evaluation functions for environmentally driven stimuli in patients.

## Data Availability

The data that support the findings of this study are available from the corresponding author upon reasonable request.

## Abbreviations

ERP: Event-related potential
DTI: Diffusion tensor imaging
fMRI: functional magnetic resonance imaging
SoA: Sense of agency
STG: Superior temporal gyrus
MTG: Middle temporal gyrus
FC: Functional connectivity
DSM-5: Diagnostic and statistical manual of mental disorders, fifth edition
EPI: Echo planar imaging
MNI: Montreal Neurological Institute
PPI: Psychophysiological interaction
FWE: Family-wise error
ROI: Region of interest
PANSS: Positive and negative syndrome scale
